# Graphite/InP and graphite/GaN Schottky barriers with electrophoretically deposited Pd or Pt nanoparticles for hydrogen detection

**DOI:** 10.1186/1556-276X-7-415

**Published:** 2012-07-23

**Authors:** Karel Zdansky

**Affiliations:** 1 Institute of Photonics and Electronics, Academy of Sciences of the Czech Republic, Chaberska 57, Prague 8, 18251, Czech Republic

**Keywords:** Graphite/InP, Graphite/GaN, Hydrogen detection

## Abstract

Large attention has been devoted worldwide to the investigation of hydrogen sensors based on various Schottky diodes. We prepared graphite semimetal Schottky contacts on polished n-InP and n-GaN wafers partly covered with nanoparticles of catalytic metals Pd or Pt by applying colloidal graphite. Metal nanoparticles were deposited electrophoretically from colloids prepared beforehand. Deposited nanoparticles were imaged by scanning electron microscopy, atomic force microscopy, and scanning tunneling microscopy on the as-made and annealed-in-vacuum samples. Current–voltage characteristics of prepared Schottky diodes had very high rectification ratios, better than 10^7^ at 1 V. It was shown that the barrier heights of these diodes were equal to the difference between the electron affinity of InP or GaN and the electron work function of the metal Pd or Pt (Schottky-Mott limit). That was a good precondition for the high sensitivity of the diodes to hydrogen, and indeed, high sensitivity to hydrogen, with the detection limit better than 1 ppm, was proved.

## Background

There has been dealing with hydrogen at many places in industry, medicine, and research and recently also for driving automotive vehicles. Hydrogen sensors in such places are needed for safety reasons because hydrogen, which cannot be detected with human senses, has a wide flammable range (4% to 75%) and its easy leakage into the environment forms a dangerous explosive of high power. Another good usage is in a device for detecting leaks in a high-vacuum apparatus. In addition, hydrogen sensors are used inside of various machineries to measure hydrogen concentration, like in various engines using hydrogen fuel. Of course, the last case requires hydrogen sensors which are stable also at high temperatures. Hydrogen monitoring is essential also in various industrial processes where hydrogen can appear via unwanted reactions with water [1].

Traditional hydrogen detectors are large and expensive, have a slow response, and require much maintenance. Hydrogen sensors based on semiconductor technology are of lower cost, smaller size, faster response, and long-term reliability. There are many different types of hydrogen sensors, which are commercially available or in development. Favored are semiconductor sensor chips to be easily integrated into electronic networks.

InP-based sensors can well operate at room temperature, and sensors based on more expensive GaN are well suited for operations at high temperatures. It is known that high-quality Schottky barriers including an effective catalytic metal like palladium (Pd) or platinum (Pt), prepared on n-type InP or n-type GaN, can detect hydrogen with high sensitivity and fast response [1]. Catalytic metals dissociate hydrogen molecules (H_2_) to atomic hydrogen (H) which is adsorbed on the semiconductor–metal interface and changes the Schottky barrier height. The concentration of the adsorbed hydrogen is proportional to the hydrogen concentration in the surrounding atmosphere; thus, the barrier height returns to the original value when hydrogen gas is removed. That is different from the case when hydrogen gas permanently changes the Schottky barrier height like in the case of Er on a p-Si Schottky diode [2]. It is generally assumed that hydrogen detection is performed in a way that the adsorbed atomic hydrogen forms a dipole layer that reduces the Schottky barrier height [3]. However, the mechanism of the dipole layer formation by hydrogen adsorption has not yet been fully clarified. A controversial explanation says that the adsorbed hydrogen is polarized by the electric field of the Schottky barrier [3].

In this paper, the electrophoretic deposition (EPD) of nanoparticles (NPs) of the catalytic metals Pd and Pt and printing colloidal graphite on n-InP and n-GaN to form Schottky barriers highly sensitive to hydrogen is reported. The paper extends our previous studies published recently [4-12].

## Methods

Pure chemicals for the preparation of catalytic metal NPs in colloid solutions were purchased from Sigma-Aldrich Corporation (St. Louis, MO, USA). One-side-polished wafers of n-InP and n-GaN were purchased from Wafer Technology (Milton Keynes, UK) and Kyma Technologies (Raleigh, NC, USA), respectively. Aqua colloid graphite of Agar Scientific (Stansted, UK) for printing Schottky contacts was purchased from Christine Gropl (Tulln, Austria).

**Figure 1:**
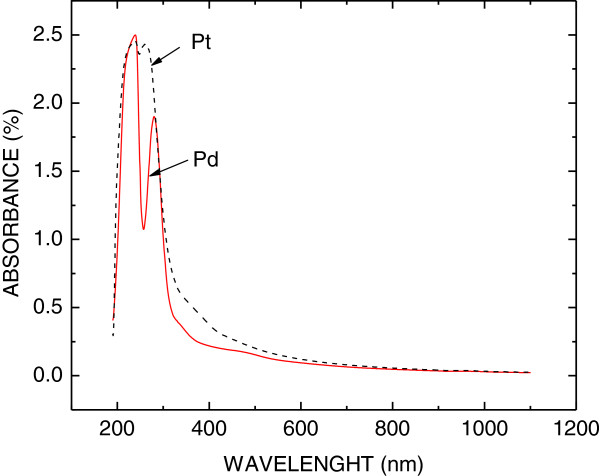
**Optical extinction spectra of colloid solutions with Pd (dashed line) and Pt NPs (full line).** The peak at 280 nm is caused by surface plasmon resonance in Pd NPs. The peak at 265 nm is caused by surface plasmon resonance in Pt NPs. The peak at 230 nm is caused by absorption in the surfactant AOT organic compound.

**Figure 2:**
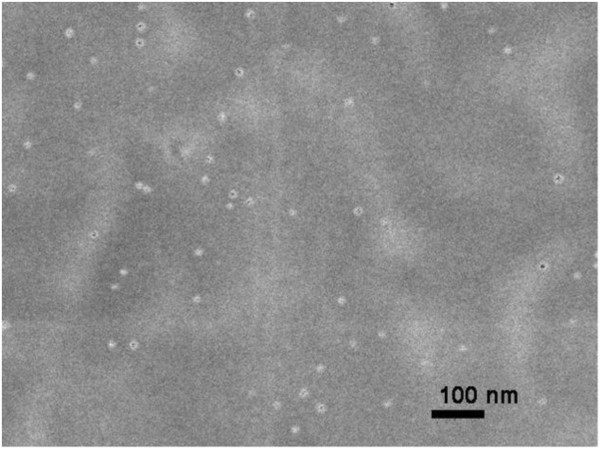
**SEM image of Pd NPs in diluted colloid solution.** Pd NPs are represented by round light spots of 10 nm in diameter. It is seen that they show no aggregates in the colloid.

Catalytic metal NPs in dioctyl sodium sulfosuccinate (AOT) reverse micelles dispersed in isooctane were prepared by the chemical reduction of metal-salt water solutions with hydrazine [13,14]. The NPs formed in the dispersion were monitored by optical extinction due to NP surface plasmon resonance, using a split-beam UV–vis spectrophotometer (SPECORD 210, Analytik Jena, Jena, Germany). In Figure 1, optical extinction spectra of two colloid solutions with Pd and Pt NPs are shown. The shape, size, and concentration of NPs were determined by scanning electron microscopy (SEM; JSM-7500F, JEOL Ltd., Akishima-shi, Japan). In Figure 2, the SEM image of the diluted colloid solution with Pd NPs is shown. It can be seen that the Pd NPs are spherical with a diameter of about 10 nm and are not aggregated.

A wafer of InP or GaN was provided with ohmic contact on the unpolished side and placed on the negative electrode (cathode) in the electrophoretic cell. The other electrode (anode) was plane-parallel with the wafer, 1 mm apart from the polished side. The cell was filled with 1 ml of prepared colloid solution with catalytic metal NPs. The EPD was performed with a negative voltage of 100 V applied on the wafer for various time periods in the range of 15 min to 4 h. The voltage was keyed with 10 Hz of frequency and 1:1 duty cycle. The layers of deposited NPs were imaged with SEM, atomic force microscopy (AFM), and scanning tunneling microscopy (STM).

**Figure 3:**
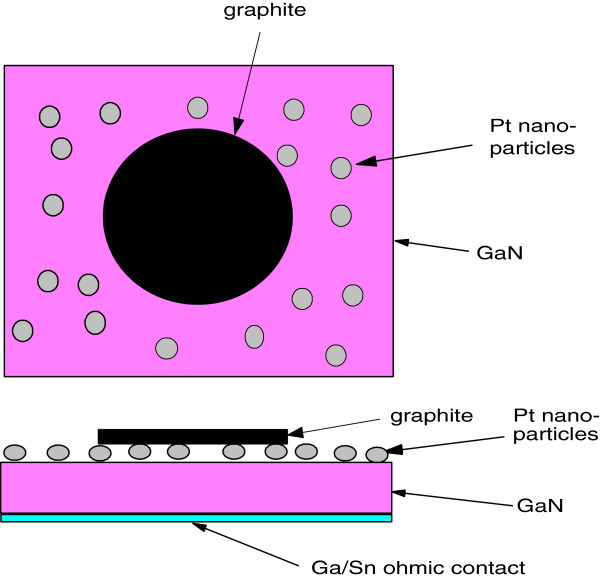
Schottky diode schema.

Schottky contacts were made on n-InP or n-GaN wafers deposited with catalytic metal NPs in a way that little beds (1 mm^2^) of colloidal graphite were printed by a blunt Teflon point and allowed to dry at room temperature. The Schottky diode schema is shown in Figure 3. Current–voltage characteristics of diodes formed with Schottky contacts and an ohmic contact on the other side of the wafer were measured using Current–voltage Source-Measure Unit 237 (Keithley, Cleveland, OH, USA) controlled using a PC with the software designed in LabView. Further, current as a function of time at constant voltage after exposing a diode to the flow of the hydrogen-nitrogen mixture was measured to determine the time response and overall sensitivity to hydrogen. When the saturation current was reached, the flow of air was switched on and the recovery of the current to the original state was measured. The response-recovery cycle was measured with various hydrogen concentrations in the range of 1 to 1,000 ppm. Two gas sources, pure nitrogen and calibrated mixture of 0.1% hydrogen in nitrogen, were mixed using gas flow meters and controllers (Bronkhorst High-Tech, Ruurlo, Netherlands) to mix flows of defined hydrogen-nitrogen mixtures.

## Results and discussion

**Figure 4:**
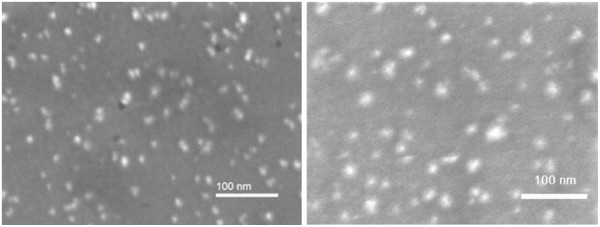
**SEM images of InP surface with Pt NPs after 1 h of EPD.** Left side: the sample was measured before annealing; right side: the sample was measured after 160°C annealing in vacuum.

**Figure 5:**
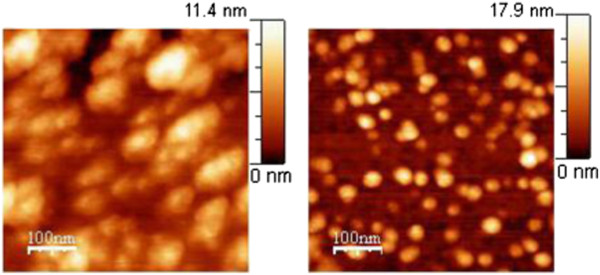
**AFM images of InP surface with Pt NPs after 1 h of EPD.** Left side: the sample was measured before annealing; right side: the sample was measured after 160°C annealing in vacuum.

Three kinds of Pt NP layers on InP were prepared by EPD with different time periods of deposition: 15 min, 1 h, and 4 h (each was four times longer). The layers were first observed by AFM and SEM, then annealed in vacuum at 160°C, and again observed by AFM and SEM for comparison. SEM images of the InP surface with Pt NPs after 1 h of EPD can be seen in Figure 4. Spherical Pt NPs are mostly separated except for some small groups formed during the EPD process. A similar image with less coverage was observed (it is not shown here) after 15 min of EPD. The coverage after 1 h of EPD was four times larger than that after 15 min of EPD, and coverage after 4 h of EPD was four times larger than that after 1 h of EPD, showing that the coverage is proportional to the EPD time period. Two SEM images of Pt NP layers after 1 h of EPD are seen in Figure 4: first, observed before annealing - on the left side; second, observed after vacuum annealing - on the right side. There is no virtual difference between these two SEM images. However, a different situation was observed by AFM. When EPD layers of Pt NPs without vacuum annealing were observed by contact-mode AFM, it was discovered that the NPs were shifted by the AFM stylus and no reproducible image was obtained, showing insufficient adhesion of NPs to the wafer surface. Only tapping-mode AFM images of unannealed samples were reproducible. Adhesion was improved by the annealing, so AFM images were the same in both contact and tapping AFM modes. In Figure 5, two AFM images of the InP surface with Pt NPs after 1 h of EPD can be seen. The size of bright spots taken on the sample before annealing (left side) is much bigger than that on the sample after annealing (right side). That is because the NPs with light coats of organic AOT molecules are sighted by AFM aside from the heavy metal cores sighted by SEM. Obviously, the coats of metal NPs are much thinner after vacuum annealing than before it. Their diameter is about 20 nm after the annealing in contrast to about 50 nm before the annealing, while the metal core diameter determined by SEM is about 10 nm. It can be explained in a way that before the annealing, the metal cores are encompassed by several coats of AOT molecules. The first coat is strongly held by chemical bonding between the Pt metal and the rest of the AOT molecules where Pt atoms substitute Na atoms of AOT. Next coats are weakly held by van der Waals forces and can be removed by annealing in vacuum.

**Figure 6:**
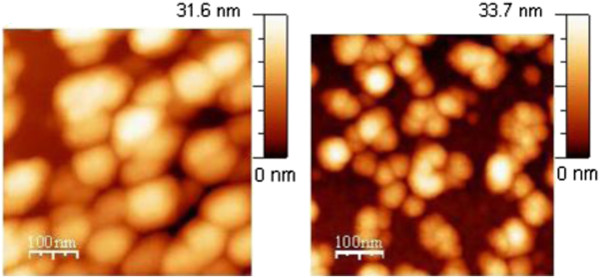
**AFM images of InP surface with Pt NPs after 4 h of EPD.** Left side: the sample was measured before annealing; right side: the sample was measured after 160°C annealing in vacuum.

SEM images of Pt NP layers prepared on InP by 4 h of EPD, before and after annealing, are again of the same kind (not shown here). The difference with respect to the 1-h EPD is that nearly all NPs are grouped forming two- or three-dimensional clusters. In the AFM images seen in Figure 6, it can be recognized whether NPs in a cluster are all aligned in one plane on the InP surface or some NPs are placed also on the top of those aligned on the surface. That is obvious when the height scales in Figure 5 are compared with the height scales in Figure 6. The maximum of the scales in Figure 5 is about 17 nm, i.e., all NPs in the image are aligned in one plane on the InP surface. The maximum of the scales in Figure 6 is about 34 nm, i.e., some NPs, the brightest ones, are placed higher, on the top of those aligned on the InP surface. It can be seen that during EPD, the first NPs are placed randomly and later, they have a tendency to be placed in contact with those which are already present on the surface.

**Figure 7:**
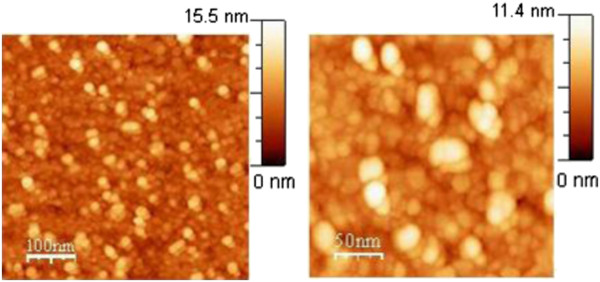
**STM images of InP surface with Pt NPs after 1 h of EPD.** Left side: the sample was annealed at 160°C for 3 min in vacuum and covered with gold layer of 9 nm in thickness by vacuum evaporation. The brightest spots represent Pt NPs while those in the background are nanograins of the gold layer. Right side: magnified STM image.

The STM measurement was not possible to be performed on the Pt NP EPD layers before vacuum annealing because of their small adhesion. To perform the STM, annealed samples were first covered with a gold layer of 9 nm in thickness by vacuum evaporation. Two STM images of Pt NPs on InP after 1 h of EPD are shown with two different magnifications in Figure 7. The STM image on the left side is similar to the AFM image shown in Figure 5 on the right side. The difference between these two images is that in the STM image, nanograins of the gold layer are also seen besides the brighter circles of Pt NPs. The STM image of twice larger magnification on the right side shows the nanograins and the Pt NPs very clearly.

**Figure 8:**
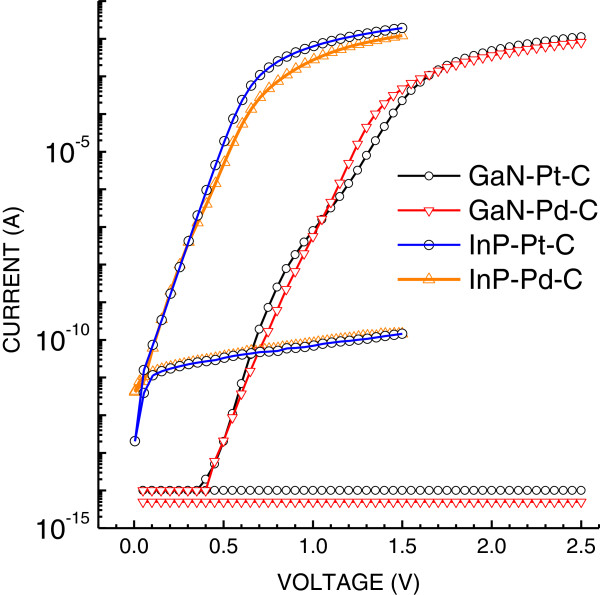
**Forward and reverse current–voltage characteristics of four types of Schottky diodes.** The type of each diode is shown in the legend. Forward currents at a voltage of 1 V are many orders of magnitude larger than the reverse ones for all diodes. Actual reverse currents of GaN diodes are smaller than the shown values whose measurements are limited by the ability of the equipment.

Forward and reverse current–voltage characteristics of Schottky diodes made by graphite on semiconductor wafers with catalytic metal NPs after 1 h of EPD are shown in Figure 8 in semi-log scale. All diodes have very high rectification ratio over 10^7^. Their ideality factors are in the region from 1 to 2 showing two involved transport mechanisms: thermionic emission and generation-recombination. The semi-linear part of each forward characteristic was used for the estimation of the corresponding Schottky barrier height, using the method described previously [11]. The estimated values of barrier heights (0.87 and 1.27 eV for InP and 0.92 and 1.42 eV for GaN with Pd and Pt) were close to vacuum level alignment between the electron work function of the metal Pd (5.12 eV) or Pt (5.65 eV) and the electron affinity of the semiconductor InP (4.38 eV) or GaN (4.10 eV). That shows a small concentration of spurious interface states and negligible Fermi level pinning - a good preposition for sensitive detection of hydrogen atoms adsorbed on the interface of the Schottky barrier. It should be mentioned that barrier heights are obviously formed by Pd or Pt contacts to InP or to GaN despite that graphite is likely to make direct contacts also. That can be explained as follows: We found that graphite Schottky barriers made without any Pd or Pt NPs show about ten times smaller currents due to larger barrier height. Therefore, graphite contacts do not assert in forward current–voltage (*IV*) characteristics of Schottky barriers with Pd or Pt NPs.

**Figure 9:**
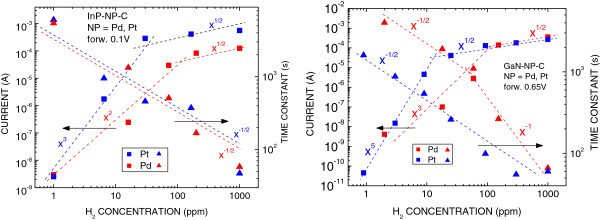
**Plots of saturated current values and response time constants as a function of hydrogen concentration.** Left side: for InP diodes; right side: for GaN diodes.

Hydrogen detection performance of the diodes was measured by the time response of current after the exposure to hydrogen. The curve of the increasing current of a forward-voltage-biased diode was measured as a function of time until the saturated current value was reached. The ratio of the saturated current value to the current value before hydrogen exposure showed the hydrogen concentration. The time period between the start of hydrogen exposure and the time when 90% of the log saturated current was reached was used as the response time constant. Plots of saturated current values and response time constants as a function of hydrogen concentration in the range of 1 to 1,000 ppm are shown in Figure 9 for the four kinds of Schottky diodes. When we observe the plots of current in Figure 9 from higher to lower hydrogen concentration *x*, we see that the current decreases with decreasing concentration as *x*^1/2^, for all four kinds of diodes. That can be explained by the process of catalytic dissociation of a hydrogen molecule to two hydrogen atoms [15]. Further down the hydrogen concentration, the current decreases faster, as *x*^*n*^, where *n* varies from 2 to 5. Mechanisms causing such faster decrease have not yet been explained. Also, the response time constant increase as *x*^−1/2^ with decreasing hydrogen concentration has yet to be explained.

The *IV* characteristics and current transients of the diodes were not changed when they were measured several months after their fabrication. Recovery transient of the diodes for switching from hydrogen to air flow consisted of two exponentials. The first exponential was fast with the time constant independent of the hydrogen concentration. The second exponential was larger and slower for Pd NPs than for Pt NPs. It can be explained by the release of hydrogen from the crystal lattice of Pd [10]. The size and shape of Pd or Pt NPs after EPD may affect the sensitivity and response times of hydrogen-sensitive diodes. We schedule studying these effects in the near future.

## Conclusions

Colloid solutions with Pd and Pt nanoparticles were prepared, and the nanoparticles were electrophoretically deposited on n-InP or n-GaN wafers. Nanoparticle layers were investigated by SEM, AFM, and STM. Schottky barriers were made on surfaces with layers of Pd and Pt nanoparticles by colloidal graphite. Prepared diodes show excellent rectification with Schottky barrier heights virtually equal to Schottky-Mott limits - a good preposition for high sensitivity to hydrogen. Indeed, it was proved that they act as very sensitive and temporally stable hydrogen sensors. Fabrication of such sensors is simple, inexpensive, and giving more sensitive devices when compared with commonly used methods. I believe that the advantage is in the protection of Pd or Pt NPs by AOT reverse micelles against chemical reactions with atoms on the semiconductor surface leading to the formation of unwanted interface states causing Fermi level pinning [16].

## Competing interests

The author declares that he has no competing interests.

## Author’s information

KZ is an emeritus scientist in the Institute of Photonics and Electronics, Academy of Sciences of the Czech Republic. He obtained his Ph.D. degree in solid state physics in 1961. His current research interests include preparation of new semiconductor nanomaterials and investigation of their electromagnetic, optical, and chemical-physical properties.
